# The Need to Unravel Arsenolipid Transformations in Humans

**DOI:** 10.1089/dna.2021.0476

**Published:** 2022-01-12

**Authors:** Teresa Chávez-Capilla

**Affiliations:** Institute of Geography, University of Bern, Bern, Switzerland.

**Keywords:** arsenic, arsenolipids, biotransformations in humans, toxicology

## Abstract

The main source of arsenic exposure to humans worldwide is the diet, in particular, drinking water, rice, and seafood. Although arsenic is often considered toxic, it can exist in food as more than 300 chemical species with different toxicities. This diversity makes it difficult for food safety and health authorities to regulate arsenic levels in food, which are currently based on a few arsenic species. Of particular interest are arsenolipids, a type of arsenic species widely found in seafood. Emerging evidence indicates that there are risks associated with human exposure to arsenolipids (e.g., accumulation in breast milk, ability to cross the blood–brain barrier and accumulate in the brain, and potential development of neurodegenerative disorders). Still, more research is needed to fully understand the impact of arsenolipid exposure, which requires establishing interdisciplinary collaborations.

The reason why arsenic is well known as the king of poisons is not surprising: arsenic is associated with a long track record of murders and has become an icon in popular culture through literature, cinema, and media sensationalism (Cullen, 2008). The reality for scientists, however, is a lot more complex. Arsenic is a widely distributed environmental pollutant that, due to both natural and anthropogenic activities, is available to humans through air, water, soil, and food (Sharma *et al.*, 2014). This ubiquity and subsequent accumulation in the food chain mean that no one is exempt from arsenic exposure. Currently, more than 200 million people worldwide are at risk of arsenic contamination from their diet (Podgorski *et al.*, 2017), with drinking water, rice, and seafood leading as the main dietary sources (de la Calle *et al.*, 2012; Naujokas *et al.*, 2013; Sharma *et al.*, 2014).

Although arsenic ranked first in the 2019 Substance Priority List (Agency for Toxic Substances and Disease Registry; Environmental Protection Agency, 2019), its health effects strongly depend on the dose and toxicity of the arsenic-containing molecules (i.e., arsenic species) of exposure.

There are already regulations worldwide to control arsenic in our diet, particularly in drinking water and rice (World Health Organization, 2010; European Commision, 2015). Arsenic contaminates drinking water through polluted surface and groundwater, where concentrations can go up to 50,000 μg L^−1^ (Singh *et al.*, 2015). These levels can have a huge impact if the affected countries have scarce remediation infrastructure. In fact, it was only after several mass poisoning events happened in America and Asia during the 20th century (Ravenscroft *et al.*, 2009; Singh *et al.*, 2015) that the World Health Organization established the maximum permitted level of arsenic in drinking water at 10 μg L^−1^, which still applies today (World Health Organization, 2010). The guidelines for food, however, are not that straightforward.

This is due to the wide range of arsenic species that we can find in nature, all of which have different toxicity (Chavez-Capilla, 2018). Some of these species resemble and replace biologically relevant molecules in essential metabolic processes, while others can irreversibly bind to enzymes, or accumulate in tissues (Ventura-Lima *et al.*, 2011). In water, we only find two arsenic species, arsenate and arsenous acid, commonly referred to as inorganic arsenic (iAs), and classified as carcinogenic (International Agency for Research on Cancer, 2012).

The arsenic species present in food are more diverse and less understood. For instance, rice accumulates up to 0.4 mg kg^−1^ of arsenic, of which 85–90% corresponds to iAs and the remaining to the methylated species methylarsonic acid and dimethylarsinic acid (Mandal and Suzuki, 2002; Carey *et al.*, 2010; Francesconi, 2010; Maher *et al.*, 2013). The carcinogenicity of these methylated arsenic species is confirmed in experimental animals, but not in humans (International Agency for Research on Cancer, 2012). Hence, the permitted maximum levels of 0.2 and 0.1 mg kg^−1^ of arsenic in rice (for adults and infants, respectively) only consider iAs (European Commision, 2015). Interestingly, although arsenic in seafood exists at concentrations between 5 and 100 mg kg^−1^ (Francesconi, 2010), only a few countries have established regulations for these foodstuffs.

In Australia and the New Zealand, the permitted maximum levels of arsenic are 1 mg kg^−1^ for seaweed and mollusks, and 2 mg kg^−1^ for fish and crustaceans (Food Standards Australia and New Zealand, 2017). In France, seaweed for human consumption can contain up to 3 mg kg^−1^ of arsenic (Centre d'Étude et de Valorisation des Algues, 2014). These limits apply to iAs, which accounts for just 10% of the total arsenic in seaweed, shellfish, fish, mollusks, and crustaceans (with the exception of Hijiki seaweed, which has mostly iAs) (Hanaoka *et al.*, 2001; Sloth and Julshamn, 2008; European Food Safety Authority, 2014). The remaining 90% corresponds to the same methylated arsenic that we can find in rice (3–46%) (Taylor *et al.*, 2017), arsenobetaine (1–70%) (Molin *et al.*, 2012; Cubadda *et al.*, 2017), arsenosugars (∼80%) (Taylor *et al.*, 2017), and arsenolipids (10–70%) (Taleshi *et al.*, 2008; Sele *et al.*, 2012).

The distribution of these arsenic species varies depending on seafood origin and type (Taylor *et al.*, 2017). For instance, while arsenosugars are predominant in seaweed, the highest percentages of arsenolipids are found in oily fish (Taleshi *et al.*, 2008; Sele *et al.*, 2012; Taylor *et al.*, 2017). None of these species is yet subjected to food safety regulations, the reason being that both arsenobetaine and arsenosugars exert low toxicity (Ohta *et al.*, 2004; Leffers *et al.*, 2013; Ebert *et al.*, 2016), and that there is little knowledge about the metabolism and impact of arsenolipids in humans.

Arsenolipids are large-sized arsenic species that resemble other naturally occurring lipids (or fats). Their existence was first suggested in the early 20th century (Sadolin, 1928); however, it was not until the 1980s that the structure of an arsenolipid was elucidated (Morita and Shibata, 1988).

Given their complex chemical structure (Fig. 1), research on these species has long been restricted due to a lack of certified standards, analytical techniques, and extraction methods. Indeed, the detection of arsenolipids requires advanced extraction procedures (Glabonjat *et al.*, 2014; Wolle and Conklin, 2018) and techniques such as high-performance liquid chromatography-inductively coupled plasma-mass spectrometry (HPLC-ICP-MS), electrospray ionization-triple quadrupole-mass spectrometry (ESI-QQQ-MS), and high resolution-mass spectrometry (HR-MS) (Francesconi, 2002; Khan *et al.*, 2016).

**FIG. 1.**
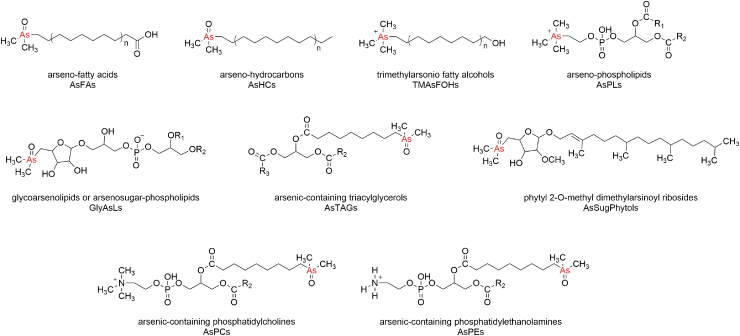
Chemical structures of different types of arsenolipids.

**FIG. 2.**
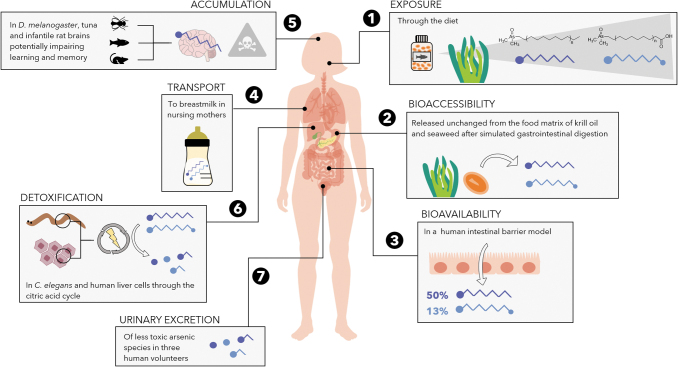
Overview of current knowledge on arsenolipid metabolism numbered from 1 to 7 according to the predicted pathway in the body overtime (human body standard license from VectorStock image 32345528; brain image retrieved from http://clipart-library.com/free/brain-clipart-transparent.html).

The use of pure arsenolipid standards is key for the successful characterization of these species. However, their synthesis is not trivial and, while no commercial standard exists, only synthetic routes to a few arsenolipids are available to date (Taleshi *et al.*, 2014; Arroyo-Abad *et al.*, 2016; Guttenberger *et al.*, 2016, 2017), and just one certified reference material has been properly characterized for arsenolipid composition (Glabonjat *et al.*, 2014). When lacking arsenolipid standards or reference materials, coupling HPLC-ICP-MS with ESI-QQQ-MS or HR-MS allows for the identification and quantification of unknown arsenic species (Madsen *et al.*, 2000; McSheehy *et al.*, 2002; Miguens-Rodriguez *et al.*, 2002; Garcia-Sartal *et al.*, 2012; Pétursdóttir *et al.*, 2018). These advanced analytical techniques are expensive and thus only available in a few laboratories worldwide.

Nonetheless, nine main structural groups of arsenolipids have been identified (Fig. 1) (Francesconi *et al.*, 1990; Rumpler *et al.*, 2008; Taleshi *et al.*, 2008; Garcia-Salgado *et al.*, 2012; Amayo *et al.*, 2013; Viczek *et al.*, 2016; Glabonjat *et al.*, 2017; Řezanka *et al.*, 2019), of which arseno-fatty acids (AsFAs) and arseno-hydrocarbons (AsHCs) are of particular interest due to their cytotoxicity, comparable to that of iAs (Meyer *et al.*, 2014, 2015a). AsFAs and AsHCs are similar to biologically relevant fatty acids (Rumpler *et al.*, 2008) and to crude oil hydrocarbons (Taleshi *et al.*, 2008), allowing them to easily accumulate in fatty tissue.

For that reason, they are the major arsenic species found in commercial fish oils (Taleshi *et al.*, 2008; Ruiz-Chancho *et al.*, 2012; Sele *et al.*, 2013; Amayo *et al.*, 2014), where arsenic levels range from 0.2 to 16 mg kg^−1^ oil (Sele *et al.*, 2012). Moreover, they are relatively stable (Khan and Francesconi, 2016), making them prone to increase in concentration as they move up the food chain (i.e., biomagnification). Although the toxicity of AsFAs and AsHCs has been shown in human liver, bladder, and brain cells (Meyer *et al.*, 2014, 2015a; Witt *et al.*, 2017a, 2017b), to fully assess their impact to human health, focusing on their bioaccessibility, bioavailability, biotransformations, and bioaccumulation is critical. Researchers have begun to investigate these processes, but more information is needed before food safety authorities can update the current guidelines.

Bioaccessibility refers to the ability of a chemical to be released from its environmental matrix and, thus, to be available to cross an organism's cell membrane. For AsFAs and AsHCs to be bioaccessible to humans, they must be released from food at the early stages of gastrointestinal digestion (i.e., mouth and stomach). The ability to then traverse the intestinal barrier and reach the bloodstream is referred to as bioavailability, which controls further access to other organs for biotransformations (e.g., metabolism and detoxification) or bioaccumulation. Both AsFAs and AsHCs can be released from krill oil and seaweed in the stomach (Chavez-Capilla, 2018), survive the conditions of the stomach and small intestine (Chavez-Capilla, 2018) (Fig. 2.2), and cross the intestinal barrier (Meyer *et al.*, 2015b).

Experiments using an intestinal barrier model found that more than 50% AsHCs and up to 13% AsFAs remain unchanged after intestinal absorption (Meyer *et al.*, 2015b), suggesting their availability to the liver and the bloodstream for distribution through the body (Fig. 2.3). These experiments, however, were performed *in vitro* and disregarded the potential role of gut microbiota on AsFA and AsHC bioaccessibility and bioavailability.

Recent research shows that the salivary microbiota can begin transforming dietary methylated arsenic and arsenosugars in the mouth (Calatayud *et al.*, 2018). Microbial-mediated metabolism continues down the gastrointestinal tract, where the nature and diversity of microbes have proven to influence the bioaccessibility and biotransformations of iAs (Lu *et al.*, 2013; Yin *et al.*, 2016, 2017). Likewise, exposure to iAs in mice alters gut microbial communities (Lu *et al.*, 2014). Given the complex chemical structure (Fig. 1) and high toxicity of AsFAs and AsHCs, the possibility of microbial-mediated transformations already occurring before intestinal absorption and the potential effects of these species on gut microbial health should not be ignored.

The bioavailability of AsFAs and AsHCs from food has been demonstrated after detecting them in the breast milk of Norwegian mothers (Stiboller *et al.*, 2017), who are generally exposed to arsenic through a fish-rich diet. Arsenic levels ranging from 0.3 to 4.46 μg kg^−1^ were found in their breast milk, where 2–61% accounted for AsFAs and AsHCs (Stiboller *et al.*, 2017). In a follow-up study, a volunteer consumed a salmon fillet containing AsHCs, of which around 3% was found in her breast milk within 24 h (Xiong *et al.*, 2020) (Fig. 2.4). Reproducing these results with a higher number of individuals is crucial, especially considering the variability of arsenic metabolism in humans (Jakobsson *et al.*, 2015), not only due to physiological and genetic factors (Tseng, 2009) but also due to gut microbial differences (McDermott *et al.*, 2020).

In this regard, a correlation between the maternal gut microbial diversity and the arsenic metabolites in breast milk after fish consumption has been observed and additional work is underway to understand this correlation (Lenters *et al.*, 2018).

The significance of these findings is irrefutable and emphasizes the need to further investigate the role of maternal gut microbiota in protecting infants from AsFA and AsHC exposure, and the impact of these arsenic species on newborn health. For instance, emerging evidence supports an association between AsHC exposure and the development of neurodegenerative disorders (Niehoff *et al.*, 2016; Müller *et al.*, 2017, 2018a; Witt *et al.*, 2017a; Zheng *et al.*, 2021). AsHCs are able to cross and disrupt the blood–brain barrier in mammals (Müller *et al.*, 2017, 2018a) and to accumulate in the brain tissue of *Drosophila melanogaster* and tuna fish (Niehoff *et al.*, 2016; Stiboller *et al.*, 2019) (Fig. 2.5).

Toxicological studies using rat brain tissues showed that AsHCs can negatively affect the mechanism underlying learning and memory in infants (Zheng *et al.*, 2021). The so-called gut–brain axis links gut microbial health to the probability of developing neurodegenerative diseases (Abughazaleh *et al.*, 2022). Therefore, it is important to investigate the role of the newborn microbiota on AsHC-induced brain damage. The presence of gut microbes in the fetus has been suggested (Younge *et al.*, 2019), raising the opportunity to also consider the contribution of prenatal microbiota in future research.

In addition, the biotransformations of AsFAs and AsHCs have only been studied using human liver cells (Chavez-Capilla, 2018; Müller *et al.*, 2018b) and *Caenorhabditis elegans* as a model organism (Bornhorst *et al.*, 2020). The main metabolites identified include less toxic arsenic species whose chemical structure suggests that, after oxidation, AsFAs and AsHCs enter the citric acid cycle (i.e., metabolic pathway responsible for energy production) (Meyer *et al.*, 2015a; Chavez-Capilla, 2018; Müller *et al.*, 2018b; Bornhorst *et al.*, 2020) (Fig. 2.6). These metabolites have been reported in human urine after the consumption of cod liver, confirming that the biotransformations of AsFAs and AsHCs respond to a detoxification mechanism (Schmeisser *et al.*, 2006a, 2006b) (Fig. 2.7).

Nonetheless, the consequences of AsFAs and AsHCs entering the citric acid cycle are not yet understood, as this could deplete cells of the energy required for essential metabolic processes (Meyer *et al.*, 2014; Müller *et al.*, 2018b). Further research on these pathways is still necessary to explain the toxic modes of action of AsFAs and AsHCs and their metabolites. The potential of gut microbiota to alter these transformations should also be studied, as it could enable the recirculation of AsFAs and AsHCs from the liver back to the small intestine (i.e., enterohepatic circulation), and subsequently distribute these toxic arsenic species in the human body (Claus *et al.*, 2016).

While there is evidence on the potential harm of AsFA and AsHC exposure to humans, a more comprehensive risk assessment is still needed for authorities to update the current regulations on arsenic in food. To date, only a few studies estimate the potential health risk of seafood consumption based on the existing evidence on AsHCs (Amin *et al.*, 2018, 2020). Not only are further toxicity tests required but also epidemiologic and metabolic studies are necessary to investigate the influence of gut microbiota on the transformations and health effects of arsenolipids.

Due to the methodological limitations hindering the proper isolation, synthesis, and analysis of most arsenolipids, current work has only focused on AsFAs and AsHCs, but all other arsenolipid species and their potential toxicity should also be researched. To conduct high-quality research on this field, scientists need pure arsenolipid standards and certified reference materials. Synthesizing new standards is essential to study additional arsenolipid species in isolation and to advance in the development of analytical methods to identify and quantify new arsenolipids in biological samples. Certified reference materials aid in validating analytical methods and ensuring reliable results.

To better understand the metabolism of arsenolipids, new *in vitro* and *in vivo* experiments are required. Gene manipulation in cell cultures has been used before to elucidate synthetic routes for some arsenic species (Xue *et al.*, 2017, 2019). Similar approaches can be used to target specific pathways of arsenolipid transformations. Moreover, performing studies in mice with different gut microbial profiles and including experiments with maternal, newborn, and/or fetal microbiomes can provide valuable knowledge on how to mitigate arsenolipids' health risks. Gaining insight into the nature of arsenolipid metabolites will aid toxicologists to focus on the right arsenic species.

Further toxicological and epidemiological studies should also include representative patterns of arsenolipid exposure in different populations and at environmentally relevant concentrations to produce data for health risk assessments. Hence, an interdisciplinary collaboration between organometallic chemists, analytical chemists, gut microbiologists, toxicologists, and epidemiologists is the way forward to successfully unravel arsenolipid transformations in humans.

## Author's Confirmation Statement

The author confirms that she alone has prepared and written the article draft.
